# The influence of culture-dependent native microbiota in Zika virus infection in *Aedes aegypti*

**DOI:** 10.1186/s13071-022-05160-7

**Published:** 2022-02-17

**Authors:** Rêgila Mello do Nascimento, Thais Bonifácio Campolina, Barbara Aparecida Chaves, Jessica Lana Fernandes Delgado, Raquel Soares Maia Godoy, Paulo Filemon Paolucci Pimenta, Nagila Francinete Costa Secundino

**Affiliations:** 1 Laboratorio de Entomologia Médica, Instituto René Rachou-FIOCRUZ-Minas, Belo Horizonte, Minas Gerais Brazil; 2 Instituto de Pesquisas Clínicas Carlos Borborema, Fundação de Medicina Tropical Dr. Heitor Vieira Dourado, Manaus, Amazonas Brazil; 3 Programa de Pós-Graduação em Medicina Tropical, Universidade do Estado do Amazonas, Manaus, Brazil; 4 Programa de Pós-Graduação em Ciências da Saúde, IRR-FIOCRUZ-Minas, Belo Horizonte, Minas Gerais Brazil

**Keywords:** Cultivable bacteria, Microbiota, Co-culture, Zika virus

## Abstract

**Background:**

Emerging and re-emerging vector-borne diseases (VBDs) pose a recurring threat to tropical countries, mainly due to the abundance and distribution of the *Aedes aegypti* mosquito, which is a vector of the Zika, dengue, chikungunya, and yellow fever arboviruses.

**Methods:**

Female 3–5 day-old *Ae. aegypti* were distributed into two experimental groups: group I—survey of cultivable bacteria; sucrose group: fed only on sucrose, i.e., non-blood-fed (UF); blood-fed group: (i) fed with non-infected blood (BF); (ii) fed with blood infected with the Zika virus (BZIKV); (iii) pretreated with penicillin/streptomycin (pen/strep), and fed with non-infected blood (TBF); (iv) pretreated with pen/strep and fed blood infected with ZIKV, i.e., gravid with developed ovaries, (TGZIKV); group II—experimental co-infections: bacteria genera isolated from the group fed on sucrose, i.e., non-blood-fed (UF).

**Results:**

Using the cultivable method and the same mosquito colony and ZIKV strain described by in a previous work, our results reveled 11 isolates (*Acinetobacter*, *Aeromonas*, *Cedecea*, *Cellulosimicrobium*, *Elizabethkingia*, *Enterobacter*, *Lysinibacillus*, *Pantoea*, *Pseudomonas*, *Serratia*, and *Staphylococcus*). *Enterobacter* was present in all evaluated groups (i.e., UF, BF, BZIKV, TBF, and TGZIKV), whereas *Elizabethkingia* was present in the UF, BZIKV, and TBF groups. *Pseudomonas* was present in the BZIKV and TBF groups, whereas *Staphylococcus* was present in the TBF and TGZIKV groups. The only genera of bacteria that were found to be present in only one group were *Aeromonas*, *Lysinibacillus*, and *Serratia* (UF); *Cedacea*, *Pantoea* and *Acinetobacter* (BF); and *Cellulosimicrobium* (BZIKV). The mosquitoes co-infected with ZIKV plus the isolates group fed on sucrose (UF) showed interference in the outcome of infection.

**Conclusions:**

We demonstrate that the distinct feeding aspects assessed herein influence the composition of bacterial diversity. In the co-infection, among ZIKV, *Ae. aegypti* and the bacterial isolates, the ZIKV/*Lysinibacillus*–*Ae. aegypti* had the lowest number of viral copies in the head-SG, which means that it negatively affects vector competence. However, when the saliva was analyzed after forced feeding, no virus was detected in the mosquito groups ZIKV/*Lysinibacillus*–*Lu. longipalpis* and *Ae. aegypti*; the combination of ZIKV/*Serratia* may interfere in salivation. This indicates that the combinations do not produce viable viruses and may have great potential as a method of biological control.

**Graphical Abstract:**

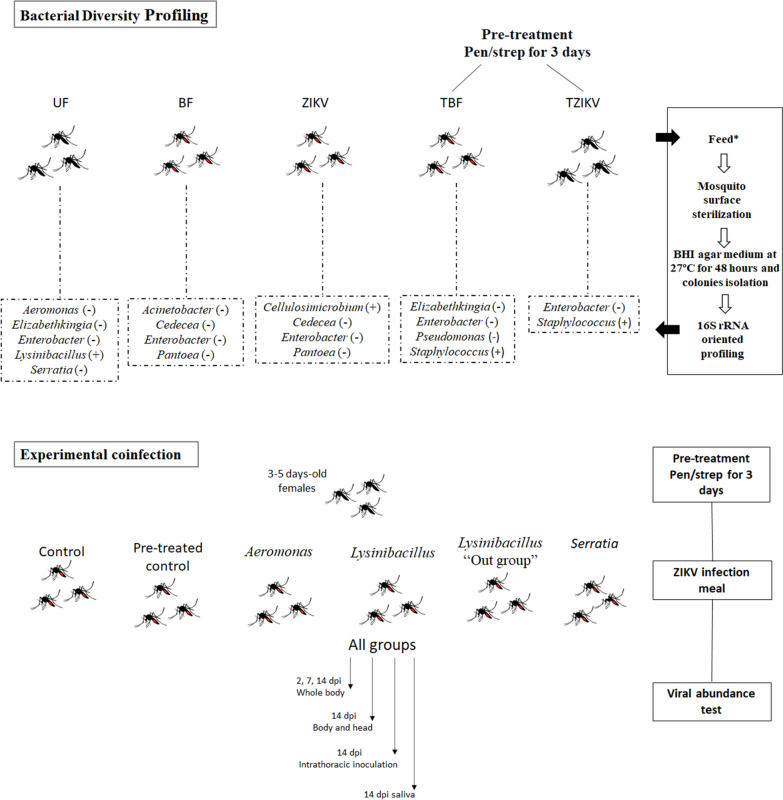

**Supplementary Information:**

The online version contains supplementary material available at 10.1186/s13071-022-05160-7.

## Background

Emerging and re-emerging vector-borne diseases (VBDs) pose a recurring threat to tropical and subtropical countries. In terms of their impact on public health and epidemiology, VBDs are responsible for 17% of all infectious diseases globally, which result in around 700,000 deaths per year [1]. The intensification of arbovirus transmission is mainly due to the abundance and distribution of the *Aedes aegypti* mosquito, a main vector of the Zika (ZIKV), dengue (DENV), chikungunya (CHIKV), and urban yellow fever arboviruses. In Brazil, despite regular vector control programs, *Ae. aegypti* persists in all states and has high urban density [2].

The principal approach for vector control of arboviruses is chemical or biological control. Chemical control focuses on the identification and elimination of breeding sites and adults [3, 4]. Biological control, i.e., the use of natural enemies, traditionally includes intervention in breeding sites. New strategies for vector control include the insecticide pyriproxyfen (PPF), which can be delivered by the insect and has the potential to block viral transmission, even in adverse scenarios [5], and the use of genetically modified endosymbionts (paratransgenesis) [6] and transgenic mosquitoes, such as the *Ae. Aegypti* strain (OX513A), which is genetically modified to prevent their offspring from developing into adults [7].

In the last few decades, advances in the study of the insect–microbiota relationship have indicated the possibility of using elements of the microbiota in vector control, with the aim of altering or using taxa with the capacity to influence the host’s physiology [8, 9]. One pioneering and successful study using this technique involved *Rhodococcus rhodnii*, an endosymbiont of *Rhodnius prolixus* and a vector of Chagas disease, which was modified to express cecropin A. This peptide results in the elimination or reduction of the number of *Trypanosoma cruzi* in the vector [10].

More recently, the bacteria of the genus *Asaia*, which was isolated from *Anopheles stephensi*, was modified to express and secrete anti-*Plasmodium* molecules capable of interfering with the activity of *Plasmodium berghei*, resulting in a significant reduction in the development of oocysts [11, 12] and a reduction in the number of pathogens ingested in the gut of insect vectors even before the end of the digestive process. Different factors are responsible for this initial reduction, such as digestive enzymes or innate response. Another possible explanation could be the action of the intracellular or extracellular bacteria in the digestive tract. Inside the midgut, pathogens and extracellular bacteria will be interacting for the first time, both competing for nutrients and survival, mainly with extracellular bacteria. As an example of intracellular bacteria, the bacterium *Wolbachia* is a known ally for VBDs. It stands out due to its ability to spread through insect populations and its effect on vector competence [13, 14].

In Culicidae, the intestinal microbiota is essential for the development and survival of the immature insect forms [15]. In addition, studies have shown that *Asaia* sp. bacteria can influence the larval development of some species of *Anopheles*, and the addition of this bacteria can accelerate the larval development. *Anopheles stephensi* larvae treated with the antibiotic rifampicin showed a delay in development and asynchrony in the appearance of posterior instars, since this antibiotic acts in the colonization and development of *Asaia* sp. [16, 17]. In another study, the genera *Enterobacter* and *Serratia* were seen to influence the digestive process due to hemolytic activity; when females of *Ae. aegypti* were treated with antibiotics (carbenicillin, tetracycline, spectinomycin, gentamicin, and kanamycin), the blood digestion was delayed. There was a change in the lysis of the red blood cells, which consequently reduced the egg production [18].

Regarding the influence of the intestinal microbiota on the biology of insects, one aspect that stands out is the ability of the microbial community to modulate infections caused by invading organisms, including pathogens transmitted by vectors to vertebrates [19, 20]. It has been shown that after treatment with antibiotics (gentamicin + penicillin–streptomycin), *Ae. aegypti* and *An. gambiae* were more susceptible to dengue virus (DENV) and *Plasmodium*, respectively [21, 22]. Villegas et al. [23] demonstrated that the families *Rhodobacteraceae* and *Desulfuromonadaceae* could be considered as biomarkers for ZIKV infection. The reintroduction of the genera *Paenibacillus*, *Proteus*, and *Chromobacterium* in *Ae. aegypti* treated with penicillin–streptomycin led to a reduction in DENV infection, suggesting that metabolites secreted by bacteria belonging to native microbiota trigger basal immune activity capable of acting against DENV infection [24, 25]. On the other hand, intestinal colonization by *Aeromonas* sp., *Escherichia coli*, and *Serratia marcescens* was associated with increased susceptibility to DENV and/or CHIKV due to the suppression of the immune response of *Ae. aegypti* [26–28]. Intestinal bacteria can also interact directly with the pathogen, for example, through the production of antimicrobial toxins, generation of free radicals, or physical blocking of receptors in the intestinal epithelium through the formation of biofilm [19, 29].

These advances in the knowledge of interactions involving microbiota, pathogen, and vector have stimulated the search for new tools to control insects and the incidence of diseases, such as arboviruses. As pathogens and bacteria share the intestinal environment, the innate ability of some microorganisms to interfere with the susceptibility of the vector could be exploited to reduce the levels of transmission of arboviruses (DENV, ZIKV, and CHIKV) and *Plasmodium* (malaria) [24, 25, 29, 30]. This study describes the culture-dependent native microbiota associated with the female *Ae. aegypti* (strain PP-Campos). Using the cultivable resident isolates in the co-infection, we explore their possible influence or interference in establishing ZIKV infection and transmission.

## Methods

### *Aedes aegypti* colony

A well-established (since 2001) Brazilian closed colony of *Ae. aegypti* (strain PP-Campos), maintained at the Laboratory of Medical Entomology, Fiocruz, Minas Gerais, Brazil, was used in this study. Mosquitoes were reared and maintained under standard insectary conditions (27 °C, 80% relative humidity, 12-h light/12-h dark photoperiod) [23].

### Study groups

Female 3–5-day-old *Ae. aegypti* mosquitoes were distributed into two experimental groups as follows:

I. Survey of cultivable bacteria; sucrose group: only fed on sucrose, i.e., non-blood-fed (UF); blood-fed group: (i) fed with non-infected blood (BF); (ii) fed with blood infected with ZIKV (BZIKV); (iii) pretreated with penicillin/streptomycin (pen/strep), and fed with non-infected blood (TBF); (iv) pretreated with pen/strep and fed with blood infected with ZIKV, i.e., gravid with developed ovaries, (TGZIKV).
II. Experimental co-infections: bacteria genera isolated from the group fed on sucrose, i.e., non-blood-fed (UF).

### Antibiotic pretreatment

Before infective feeding, the mosquitoes were treated for three consecutive days with a 10% sterile sucrose solution containing 50 U/ml penicillin and 50 µg/ml streptomycin (pen/strep) ad libitum to remove or clear part of the native microbiota. The other groups were maintained for the same period in a 10% sterile sucrose solution. In all, about 200 mosquitoes were used in each group.

### Zika virus strain

Brazilian human isolate of ZIKV from the state of São Paulo, the strain ZIKV/*H*. *sapiens*/Brazil/SPH/2015, was used in all the experiments [31]. The stocks were propagated in an *Aedes albopictus* cell line (C6/36) and grown in Leibovitz L-15 medium supplemented with 2% inactivated fetal bovine serum, 20 μg/ml gentamicin, 5 μg/ml amphotericin B, and 200 U/ml penicillin [32]. Virus titration followed the 50% tissue culture infectious dose method [33].

### Experimental infection

For 3 days before the infective meal, 3–5-day-old female *Ae. aegypti* mosquitoes were pretreated with 50 U/ml penicillin plus 50 μg/ml streptomycin in the sucrose meal (10% ad libitum sucrose solution). The mosquitoes were infected via a membrane-feeding assay using a glass-feeding device that was filled with 400 µl of mouse blood with or without 1/3 of ZIKV (a titer of 1 × 10^5^ plaque-forming units (PFU)/ml). The mosquitoes were allowed to feed for 1–2 h on the blood meal. After feeding, approximately 200 fully engorged females were separated into new cages and maintained on 10% sucrose solution ad libitum for up to 2, 7, and 14 days post-infection (dpi). Using real-time polymerase chain reaction (qPCR), as described below, 100% infection was determined on the seventh dpi in the groups used to survey cultivable bacteria.

### Bacterial diversity profiling using a culture-dependent technique

The mosquitoes were first immobilized at –20 °C for a few seconds and their surfaces sterilized by a 1-min submersion in 1% hypochlorite and 15–30 s in 70% ethanol, and then rinsed three times with phosphate-buffered saline (PBS) [34]. Subsequently, a pool of 10 midguts from each group was dissected under laminar flow, and bacterial members of the microbiota within each pool were isolated in tubes containing 200 µl of brain–heart infusion broth (BHI) (Sigma, St. Louis, MO, USA), which is a non-selective medium for promoting the growth of an ample range of bacteria. The pools were gently macerated using a microtube homogenizer system. A subsample of the homogenate (100 µl) was then pour-plated in the BHI agar medium at 27 °C for 48 h. The observed colonies, differentiated by color and morphological characteristics, were divided according to color, elevation, and shape, and were subjected to the spread plate technique in agar medium three times. The pure colonies expanded in the liquid medium, and each of the isolates, were differentiated by Gram staining and taxonomic profile. Finally, after the in vitro assays, the bacteria were re-plated and re-sequenced to ensure that the bacterial genera remained the same.

### 16S rRNA-oriented profiling

A total of 52 bacterial isolates were obtained and the bacterial genomic DNA (gDNA) extraction was performed with the DNeasy Blood & Tissue kit (Qiagen, Hilden, Germany) according to the manufacturer’s instructions. The genomic material was quantified and its purity assessed using a spectrophotometer (NanoDrop, Thermo Fisher Scientific). Bacterial gDNA from each sample served as a template for a PCR assay using Illustra PuReTaq Ready-To-Go PCR beads (GE Healthcare, Buckinghamshire, UK) and the primers 16S ribosomal RNA 27 sense 5′-AGAGTTTGATCA/CTGGCTCAG-3′, and 1492 antisense 5′-TACGGT/CTACCTTGTTACGACTT-3′. Amplification conditions were a 96 °C hold for 2 min, 30 cycles of 95 °C for 1 min, 50 °C for 1 min, and 72 °C for 3 min, followed by 5 min at 72 °C. Amplified products were visualized on a 1% agarose gel (Fisher Bioreagents, NH, USA) and cleaned using the Wizard SV Gel and PCR Clean-up System (Promega, WI, USA). Twenty nanograms of the purified PCR product was sequenced using the PCR primers described above and the DYEnamic ET Terminator on a DNA sequencer (ABI 3730, Life Technologies, CA, USA). Sequences were aligned, merged into contigs, and trimmed using Sequencer software (version 5.4.6). Resulting sequences were analyzed for similarity using the sequence analysis tool RDP (Ribosomal Database Project- Update 5) and checked against the NR database (Non-redundant, NCBI database) using the BLASTN algorithm with default parameters [35]. The best BLAST hit was selected considering a 97% identity threshold. Taxonomic profiling of bacterial communities using 16S rRNA sequences as a target has some well-known limitations, including database bias (e.g., due to a small amount of mosquito associated sequences deposited). The differences in each of the 16S rRNA variable regions are exhibited when resolving taxonomic levels [36, 37], and even cross-kingdom amplification [38], amongst other issues [39, 40]. For caution, we chose to report taxonomic identifications at the genus level only.

### Co-infection: bacterial isolates and ZIKV

The bacterial genera isolated from the group fed on sucrose (UF) were used in the co-infection. As an “out-group,” we used *Lysinibacillus* isolated from the midgut of *Lutzomyia longipalpis*. The females were previously treated with pen/strep as described above, and experimentally co-infected with ZIKV and 1 × 10^8^ colony-forming units (CFU)/ml of the bacterial isolates. As a control group, untreated mosquitoes and mosquitoes pretreated with pen/strep were used and evaluated until the 14th dpi.

### Real-time qPCR for ZIKV detection and quantification

Entire mosquito bodies and head-SGs were dissected from the experimental groups at 2, 7, and 14 dpi. These mosquito tissues were macerated and processed separately for RNA extraction (QIAamp Viral RNA Mini Kit, Qiagen, Hilden, Germany). Specific ZIKV primer and probe sets for were designed, as previously described by Lanciotti [41]. Primers were synthesized by Integrated DNA Technologies and probes with 5-FAM used as the reporter dye (Thermo Fisher). The number of viral copies was determined by automatic comparison with the specific set of standard samples. All real-time assays were performed using the TaqMan RNA-to-Ct 1-Step Kit, with amplification in the 7500 Fast and 7500 real-time PCR system, according to the manufacturer’s protocol.

### Intrathoracic inoculation of infected salivary glands

In parallel with the co-infection experiments, 14 days after the ZIKV-infective blood meal, 10 *Ae*. *aegypti* mosquitoes from each co-infective group were quickly killed by cold exposure. The salivary glands (SGs) were dissected, and the SGs ground with pestle tips in 10 µl of L15 media (without antibiotics). Subsequently, the homogenates were used for the intrathoracic inoculation of 10 naïve 3–5-day-old *Ae*. *aegypti* (Nanoinject II, Drummond Scientific Co., Broomal, PA, USA). Post-inoculation, these mosquitoes were maintained on 10% sucrose solution ad libitum for 7 days and processed via qPCR for ZIKV quantification [42].

### Saliva collection and detection of ZIKV

The saliva from individual mosquitoes, co-infected through membrane feeding, were collected at 14 days after infection with ZIKV and the bacteria. In order to verify the effect of the bacterial isolates directly in the virus, *Ae*. *aegypti* were anesthetized with CO_2_ and kept on an ice plate while the wings and legs were removed. The proboscis of each mosquito was inserted into a 10 µl pipette tip containing a 1:1 solution of 5 µl sterile fetal bovine serum and 30% sucrose solution. After 1 h, the contents of the tips were collected in 1.5 ml tubes and stored at –70 °C until processing. ZIKV in mosquito saliva was quantified using qPCR [43].

### Statistical analysis

The results were analyzed using the GraphPad Prism 7 program. The non-parametric Kruskal–Wallis test (ANOVA) was used for statistical analysis. Values of *P* < 0.05 were considered significant (*P < 0.05; **P < 0.001; ***P < 0.0001; or ****P < 0.00001).

## Results

### Cultivable native microbiota of *Ae. aegypti*

In 2018, Villegas et al. [23] revealed aspects of the profile of the native bacterial community of *Ae. aegypti* (strain PP-Campos) using 16S amplicon sequencing (culture-independent method). Using the cultivable method and the same mosquito colony and the ZIKV strain described by Villegas, our results revealed 11 isolates (*Acinetobacter*, *Aeromonas*, *Cedecea*, *Cellulosimicrobium*, *Elizabethkingia*, *Enterobacter*, *Lysinibacillus*, *Pantoea*, *Pseudomonas*, *Serratia*, *Staphylococcus*). General aspects of the colonies presented a white color, circular shape, entire margin, and convex elevation, and eight were Gram-negative (72.7%) and three were Gram-positive (27.3%) (Table 1).

**Table 1:** Genera of bacteria isolated from different physiological conditions of *Ae. Aegypti*

Genus (Gram)	UF	BF	BZIKV	TBF	TGZIKV
*Acinetobacter* (−)		X			
*Aeromonas* (−)	X				
*Cedecea* (−)		X			
*Cellulosimicrobium* (+)			X		
*Elizabethkingia* (−)	X		X	X	
*Enterobacter* (−)	X	X	X	X	X
*Lysinibacillus* (+)	X				
*Pantoea* (−)		X			
*Pseudomonas* (−)			X	X	
*Serratia* (−)	X				
*Staphylococcus* (+)				X	X

(+) Gram-positive and (−) Gram-negative. Groups: UF: fed on sucrose, non-blood-fed; BF fed with non-infected; BZIKV: fed with blood infected with ZIKV; TBF: pretreated with pen/strep and fed with non-infected blood; TGZIKV: pretreated and fed with blood infected with ZIKV, i.e., gravid with developed ovaries

**Fig. 1:**
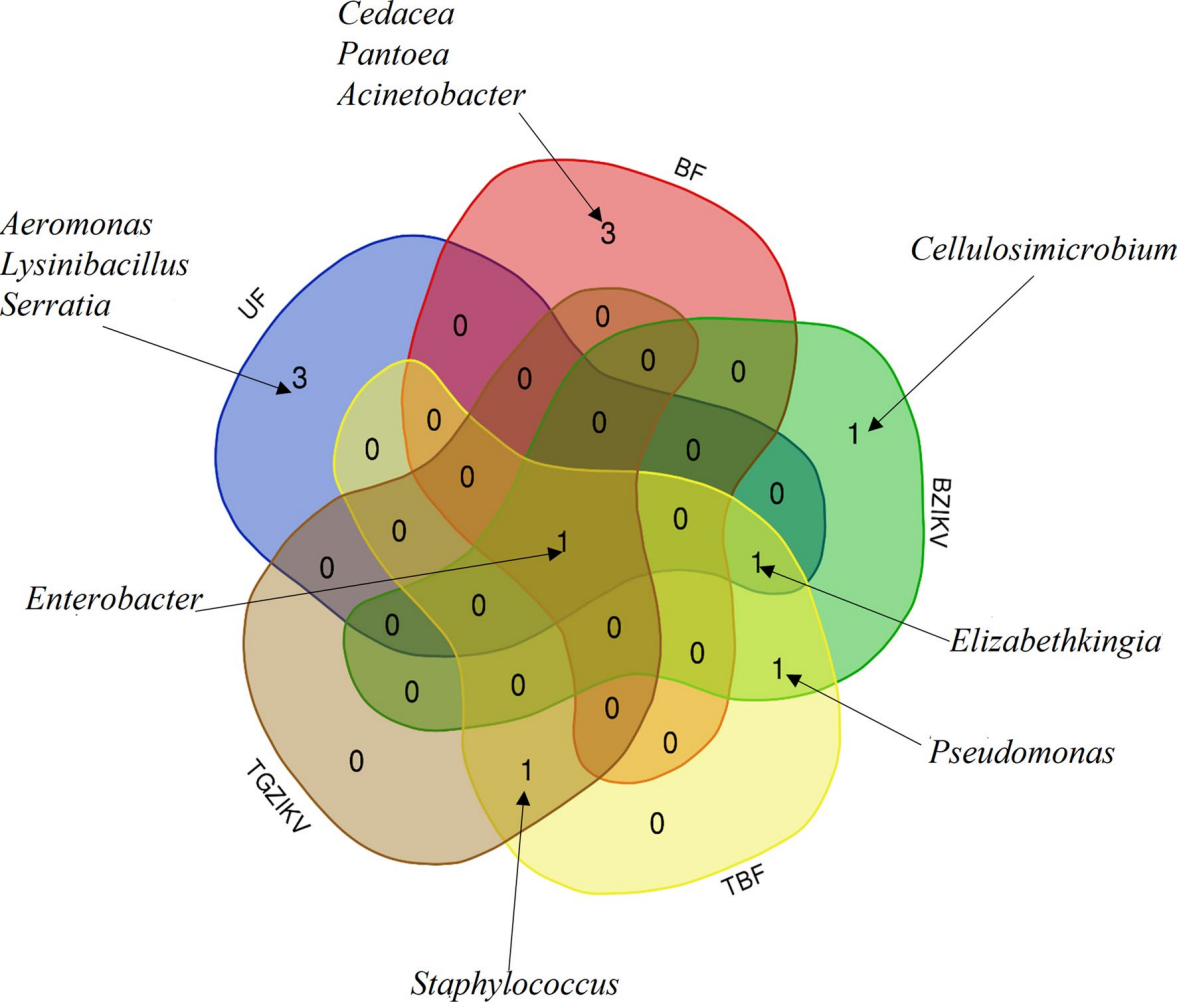
Exclusive and associated bacterial genera identified in *Aedes aegypti* (strain PP-Campos). Diagram generated with the Venn online tool (http://bioinformatics.psb.ugent.be/cgibin/liste/Venn/calculate_venn.htpl)

The analysis of the groups above allowed us to observe the following distribution of bacterial isolates: (1) *Enterobacter* present in all evaluated groups (UF, BF, BZIKV, TBF, and TGZIKV); (2) *Elizabethkingia* present in groups UF, BZIKV, and TBF; *Pseudomonas* present in groups BZIKV and TBF; and *Staphylococcus* in groups TBF and TGZIKV. The unique (exclusive) bacteria genera that were present in only one group were *Aeromonas*, *Lysinibacillus*, and *Serratia* (UF); *Cedacea*, *Pantoea*, and *Acinetobacter* (BF); and *Cellulosimicrobium* (ZIKV) (Fig. 1).

### Effect of bacterial isolates in ZIKV infection

Based on the isolates *Aeromonas*, *Elizabethkingia*, *Enterobacter*, *Lysinibacillus*, and *Serratia* revealed in the UF group, three isolates were selected according to their effect, as follows: *Aeromonas*, associated with *Ae. aegypti*, which increased susceptibility to infection by DENV-2 [28]; *Lysinibacillus*, whose species *Lysinibacillus sphaericus* produces a toxin used as a larvicide in the control of *Culex* and *Anopheles* spp. [44]; *Serratia*, which is described as having anti-*Plasmodium* activity by stimulating the activation of the Toll receptor pathway in *An. stephensi* [45] and in *Ae. aegypti* increases its susceptibility to DENV infection [46].

An isolate from another insect vector out-group *Lysinibacillus*–*Lu. longipalpis* was used due to its modulation in the course of infection by *Leishmania* [47], and this genus was found in our mosquito. We also evaluated whether these isolates were able to positively or negatively modulate ZIKV infection.

Whole insects were co-infected with four isolates and analyzed 2, 7, and 14 dpi. Note that there was still the presence of blood on the second day; however, *Aeromonas* and *Lysinibacillus* from Lu. *Longipalpis* and *Serratia* showed a twofold median reduction. This reduction in viral abundance may result in the completion of nutrients (bacteria and viruses) in the blood meal. At 7 dpi, when the digestive process was finished, we observed intense viral colonization, with a median of around 10^7^, and at 14 days, we observed a reduction in viral abundance. We conclude that it is difficult to determine any bacterial action concerning the virus using the entire insect.

**Fig. 2:**
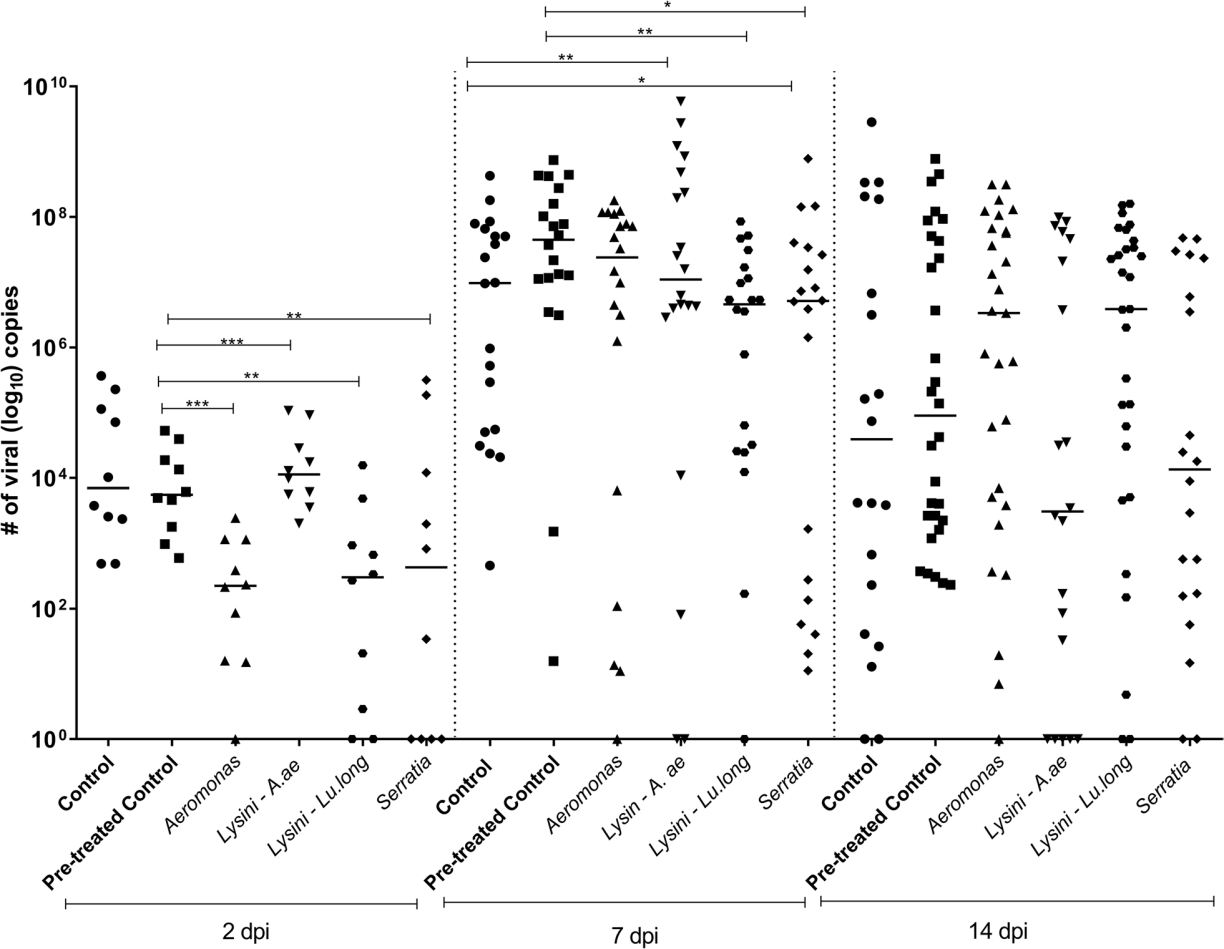
Experimental co-infection assay showing the number of copies of ZIKV cDNA in the entire mosquito of co-infected mosquitoes at 2, 7, and 14 days after the infectious blood meal. Statistically significant differences were observed on the second day between controls and mosquitoes co-infected with *Aeromonas*, *Serratia*, *Lysinibacillus*–*Ae. aegypti*, and *Lysinibacillus*–*Lu. longipalpis.* On the seventh day, there was a statistical difference between the control groups and those co-infected among all groups. On the 14th day, there was no statistically significant difference. *Lysinibacillus*–*Ae. aegypti* = Lysini–A. ae, and *Lysinibacillus*–*Lu. longipalpis* = Lysini–Lu. long

The mosquitoes were simultaneously co-infected with bacteria and ZIKV, which was mixed into normal blood. On the second dpi, it was possible to observe a significant difference in the number of viral copies per mosquito between the control groups (treated with antibiotics and untreated) and the groups co-infected by ZIKV/*Aeromonas* and ZIKV/*Lysinibacillus*–*Lu. longipalpis* (out-group), On the seventh day, we observed the maintenance of the statistical difference between the control group and the group co-infected with ZIKV/*Lysinibacillus–Lu. longipalpis*. We also observed a difference between the control group that was treated and the group that was co-infected with ZIKV/*Serratia*. On the last day (14 dpi), we did not observe any differences in the intensity of the infection (Fig. 2).

The infection rate on the second dpi for the control groups (treated and untreated) and the group co-infected with ZIKV/*Lysinibacillus*–*Ae. aegypti* was 100%. For ZIKV/*Aeromonas*, it was 90%; for ZIKV/*Lysinibacillus–Lu. longipalpis*, it was 80%; and for ZIKV/*Serratia*, it was 60%. On the seventh dpi, the infection rate for the control groups (treated and untreated) and the co-infected ZIKV/*Serratia* group was 100%; for the ZIKV/*Aeromonas* and ZIKV/*Lysinibacillus*–*Lu. longipalpis* groups, it was 95%; and for the co-infected ZIKV/*Lysinibacillus–Ae. aegypti* group, it was 90%. On the 14th dpi, the infection rate for the control (treated) group was 100%; for the co-infected ZIKV/*Aeromonas*, it was 96%; for the control (untreated), co-infected ZIKV/*Lysinibacillus*–*Lu. longipalpis* and ZIKV/*Serratia* group, it was 90%; and for the co-infected ZIKV/*Lysinibacillus*–*Ae. aegypti* group, it was 75% (Fig. 2).

In summary, the experimental co-infection assay was successful in all groups when analyzing the entire mosquito of co-infected mosquitoes at 2, 7, and 14 dpi. At the beginning of the infection (second dpi), we observed the smallest median numbers (10^2^ to 10^4^) in cDNA viral copies. Statistically significant differences were observed in mosquitoes co-infected with *Aeromonas* and *Lysinibacillus*–*Lu. longipalpis* (**P = 0.001, ***P = 0.0001). On the seventh day, after the digestive process, it was possible to note a higher number (10^7^) of copies in those co-infected with *Lysinibacillus* from *Ae. aegypti*, and *Lu. longipalpis* and *Serratia* (*P = 0.05, **P = 0.001). However, on the 14th day, we noticed a small reduction.

### Impact of co-infection in the body and head-SG

No positive or negative changes in viral abundance were observed on the 14th dpi.

Since we did not observe any viral abundance using the whole insect, we performed a second infection using isolates identical to those described above and analyzed the body and head-SG. The samples were analyzed 14 days after the intrinsic incubation. We observed medians of around 10^7^ and a smaller number of copies in the head-SG. In summary, we can assume that after colonization of the body, there was a reduction in the invasion of the gland, perhaps indicating some effective barrier related to the infection of the salivary gland.

Therefore, we wondered whether there was any barrier or variation regarding the abundance of infection in the target organs of the mosquito. In all the groups, we observed greater viral abundance in the entire mosquito (Fig. 2).

**Fig. 3:**
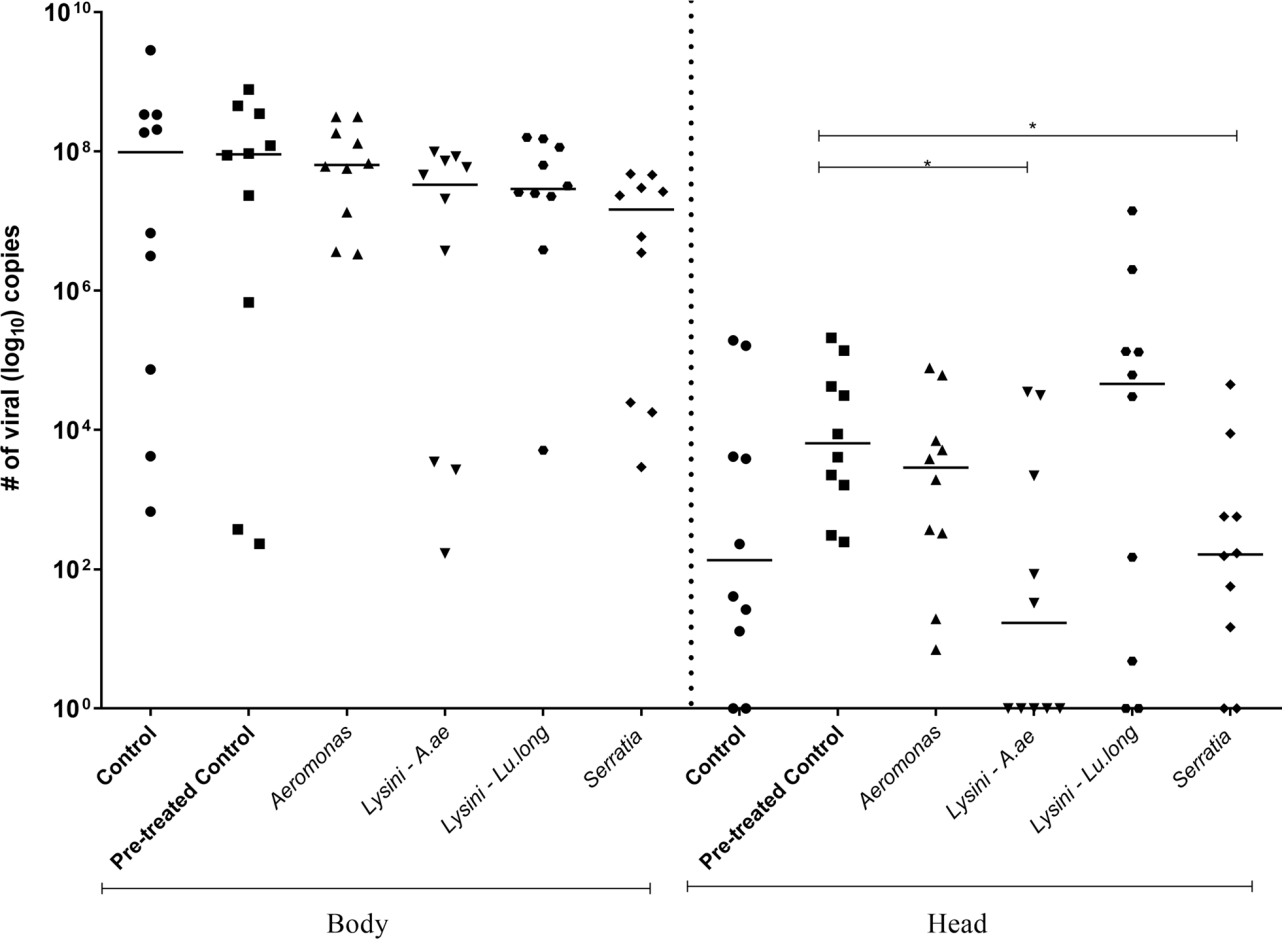
Experimental co-infection assay showing the number of copies of ZIKV cDNA in the head-SG and body 14 days after feeding with co-infected blood. When comparing the number of viral copies in the body, we did not observe any differences among the groups. When comparing the groups in the head-SG, statistically significant differences were observed between the control treated with pen/strep and the groups co-infected with *Lysinibacillus*–*Ae. aegypti* and *Serratia* (*P = 0.05). *Lysinibacillus*–*Ae. aegypti* = Lysini–A. ae, and *Lysinibacillus*–*Lu. longipalpis* = Lysini–Lu. long

In summary, the infection rate was 100% in the head-SG for the control group (treated) and for the co-infected ZIKV/*Aeromonas*. For the control group (untreated), the ZIKV/*Lysinibacillus–Lu. longipalpis* co-infected group, and the co-infected ZIKV/*Serratia* group, infection was 80%, and for the co-infected ZIKV/*Lysinibacillus–Ae. aegypti* group, it was 50%. In the body, the infection rate for the control groups (treated and untreated) and the co-infected ZIKV/*Lysinibacillus*–*Ae. aegypti*, ZIKV/*Lysinibacillus*–*Lu. longipalpis* and ZIKV/*Serratia* groups was 100%, and for the co-infected ZIKV/*Aeromonas* group, it was 90% (Fig. 3).

Interestingly, the group with the highest median number of viral copies in the head-SG was the out-group that was co-infected with *Lysinibacillus*–*Lu. longipalpis*, and the group with the lowest number of viral copies was the one co-infected with *Lysinibacillus*–*Ae. aegypti* (Fig. 3). Our results suggest that *Lysinibacillus*–*Ae. aegypti* positively influenced the reduction in viral load compared to the group co-infected with *Lysinibacillus*–*Lu. longipalpis.*

### Poof of viability of ZIKV after mosquito infection

To assess the viability of ZIKV in co-infection (i.e., the capacity of the virus to reach the salivary gland and be transmitted), we analyzed the viral abundance on the 14th dpi (Fig. 3).

To avoid misinterpretation due to quantification of numbers of viral copies in the salivary glands (i.e., quantifying genetic material), a subset of mosquitoes from the second infection were used in the intrathoracic injection, and we analyzed whether the virus was able to establish a new infection.

The intrathoracic injection serves to avoid the midgut barrier and facilitate the infection, and the mosquitoes were analyzed at 7 dpi and presented 30–50% infection using *Aeromonas*, *Lysinibacillus*, and *Serratia* and 100% for *Lu. longipalpis*.

Additionally, the salivary glands of the mosquitoes were dissected, and the homogenate was inoculated into the thorax of naïve mosquitoes. At 7 days after the intrathoracic infection, it was possible to observe the viral load in these mosquitoes. The infection rate for the ZIKV/*Lysinibacillus*–*Lu. longipalpis* co-infected group was 100%; for the control (treated) group, it was 80%; for the co-infected ZIKV/*Serratia*, it was 70%; and for the control groups (untreated), co-infected ZIKV/*Aeromonas*, and co-infected ZIKV/*Lysinibacillus*–*Ae. aegypti* groups, it was 50%.

**Fig. 4:**
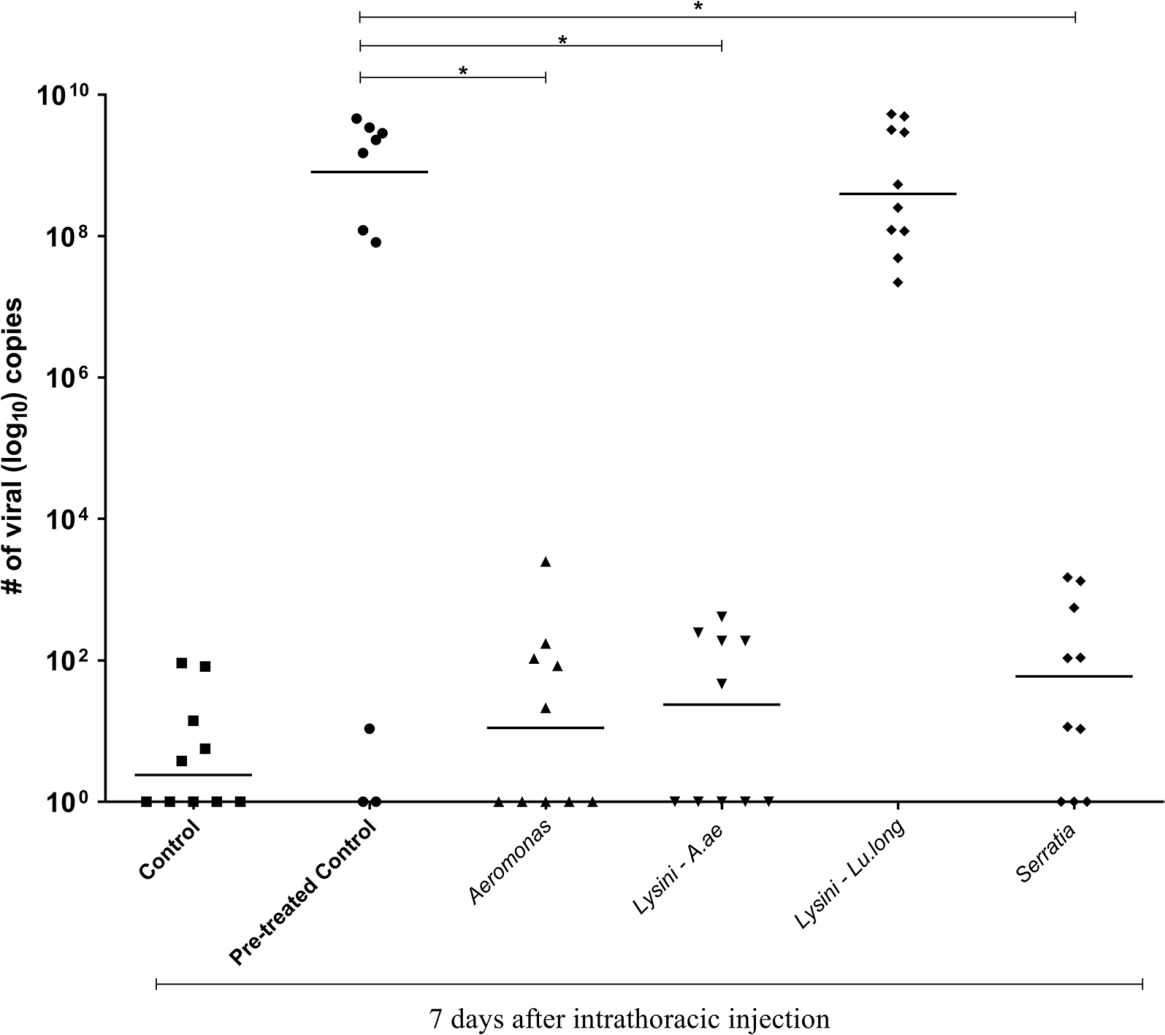
Experimental assessment of the viability of ZIKV in co-infection. The infected salivary gland homogenates from mosquitoes analyzed for viral abundance on 14 dpi were inoculated by intrathoracic injection in naïve mosquitoes, and the viral load was analyzed 7 days after infection. Statistically significant differences were observed for most groups when comparing the number of viral copies of cDNA from co-infected mosquitoes with controls (*P = 0.05, ****P = 0.00001). *Lysinibacillus*–*Ae. aegypti* = Lysini–A. ae, and *Lysinibacillus*–*Lu. longipalpis* = Lysini–Lu. long

The number of copies of ZIKV in the control group treated with antibiotics was higher than that in the control group (untreated) and was similar in the group co-infected with ZIKV/*Lysinibacillus*–*Lu. longipalpis*, with a median of around 10^9^ viral copies per mosquito. In the other groups co-infected with ZIKV/*Aeromonas*, ZIKV/*Lysinibacillus*–*Ae. aegypti*, and ZIKV/*Serratia*, the number of copies of the ZIKV was approximately 10^2^ copies of cDNA per mosquito (Fig. 4). Our results show that in all the injected groups, the virus remained viable at 7 dpi.

### The influence of co-infection on transmission

**Fig. 5:**
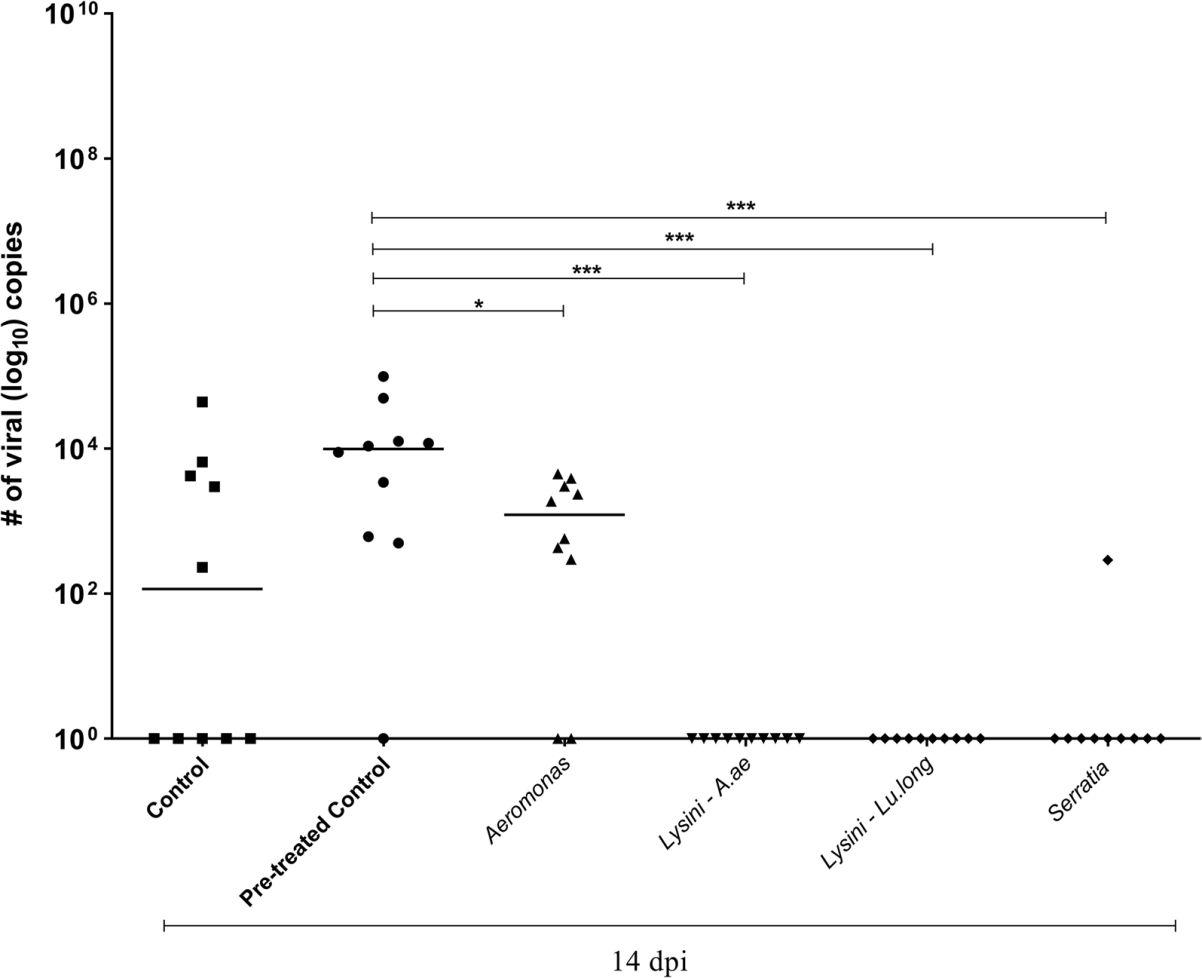
Number of ZIKV cDNA copies in the saliva of mosquitoes infected 14 days after feeding with co-infected blood. Statistically significant differences were observed for all groups when comparing the number of viral copies of cDNA from the co-infected mosquito saliva with the treated control (*P = 0.05, ***P = 0.0001). Against the untreated control, statistically significant differences were observed in the co-infection with *Lysinibacillus*, isolated from *Aedes aegypti* and *Lu. longipalpis* (*P = 0.05). *Lysinibacillus*–*Ae. aegypti* = Lysini–A. ae, and *Lysinibacillus*–*Lu. longipalpis* = Lysini–Lu. long

Concomitantly, to assess the impact of co-infection on transmission, the saliva of part of the mosquitoes that were infected was analyzed. Salivation revealed a median of around 10^4^ copies in the control group pretreated with pen/strep and around 10^3^ in the group co-infected with ZIKV/*Aeromonas*. It was not possible to detect ZIKV in the other groups analyzed (Fig. 5). In summary, the virus was not detected by qPCR in saliva, which could have an impact on transmission—i.e., mosquitoes co-infected with ZIKV/*Lysinibacillus*–*Ae. aegypti*, ZIKV/*Lysinibacillus*–*Lu. longipalpis* cannot transmit viable viruses—and in ZIKV/*Serratia,* only one was positive, which implies that *Serratia* may also interfere in transmission, even though viruses in the head were detected, as shown in Fig. 3, which influences vector competence.

## Discussion

Zika fever is an emerging systemic arboviral disease caused by ZIKV. Vector transmission, more specifically mosquitoes of the genus *Aedes*, is the virus’s primary mode of transmission to the host [48, 49]. The first autochthonous case of ZIKV infection in the Americas was reported in northeastern Brazil in May 2015. Subsequently, Zika spread rapidly throughout Brazil, reaching its peak in 2016 with 273,904 reported cases [50–52]. The transmission of ZIKV persists, although the number of cases has decreased since 2018 (19,020 reported cases) [51]. Areas with competent vectors are at risk of re-emergence of ZIKV, making advances in the search for new strategies for vector control essential. Knowledge regarding the microbiota associated with mosquitoes of the genus *Aedes* is fundamental in order to understand the existing interaction with the pathogen and elucidate its impact on vector competence.

Mosquitoes host a diverse community of microorganisms that can influence development [15], reproduction [18], and susceptibility to pathogens [53]. In this study, using a cultivable bacteria method, we identified the microbiota resident in *Ae. aegypti* (Cepa PP-Campos) under distinct feeding aspects. We isolated 11 bacterial genera, of which eight were Gram-negative (72.7%) (*Acinetobacter*, *Aeromonas*, *Cedecea*, *Elizabethkingia*, *Enterobacter*, *Pantoea*, *Pseudomonas*, and *Serratia*) and three were Gram-positive (27.3%) (*Cellulosimicrobium*, *Lysinibacillus*, and *Staphylococcus*), and these were distributed in five experimental groups (UF, BF, BZIKV, TBF, and TGZIKV). These findings are in agreement with those of Villegas et al. [23] who, through the use of high-throughput sequencing, found Gram-negative bacteria to be predominant in mosquitoes fed sucrose and/or blood and infected with ZIKV.

The bacterial diversity showed a change according to the distinct feeding aspects evaluated. As shown in our results (Table 1), our analysis among the experimental groups revealed four genera (*Enterobacter*, *Elizabethkingia*, *Pseudomonas*, and *Staphylococcus*) associated between the groups. *Enterobacter* was identified in all experimental groups (UF, BF, BZIKV, TBF, and TGZIKV). In addition, it is already considered the prevalent genus of intestinal bacteria cultivable in *Ae. aegypti* and *Ae. albopictus* [54], and other studies have described its ability to deal with oxidative stress caused by feeding with blood [55] and its association in the blood digestion process [18, 56]. *Elizabethkingia*, identified in the UF, BZIKV, and TBF groups, has also been described in *Ae. aegypti* fed with sucrose [57] and in the groups fed with sucrose, fed with blood, fed with blood infected with ZIKV, and gravid mosquitoes infected with ZIKV that were described by Villegas et al. [23], and represented at the family level (Flavobacteriaceae). *Pseudomonas*, identified in the BZIKV and TBF groups, are present in *Ae. aegypti* evaluated according to different food sources [23, 53, 58, 59]. *Staphylococcus* was identified in the TBF and TGZIKV groups. Yadav et al. [54, 60] isolated the *Staphylococcus* genus from emerged *Ae. aegypti* mosquitoes [51] and *Ae. albopictus* fed on blood. Additionally, the Staphylococcaceae family was described in the groups (fed with sucrose, fed with blood, fed with blood infected with ZIKV, and gravids infected with ZIKV) that were evaluated by Villegas et al. [23].

Some bacterial isolates were unique to only one experimental group, such as *Aeromonas*, *Lysinibacillus*, and *Serratia*, which were present in the UF group. *Aeromonas* has been described in *Ae. aegypti* [24], *Ae. albopictus* [60], *Culex quinquefasciatus* [61], and *An. gambiae* [62]. The genus *Lysinibacillus* was isolated from emerged *Ae. aegypti* and *Ae. albopictus* adults [54] and pupa and adults of *Lu. longipalpis* [47]. *Serratia* was isolated from *Ae. aegypti* by Apte-Deshpande et al. [27], who identified the species *Serratia odorifera* in the midgut of larvae and females of colonized mosquitoes.

*Cedecea*, *Pantoea*, and *Acinetobacter* were identified only in the BF group. Gusmão et al. [56] also isolated *Cedecea* from the midgut of *Ae. aegypti* fed on blood (culture-independent method). *Acinetobacter* was shown to be frequently associated with different species of mosquitoes, including *Ae. aegypti*, *Ae. albopictus*, *Ae. triseriatus*, *An. stephensi*, *Cx. pipiens*, *Cx. quinquefasciatus*, and *Psorophora columbiae* [63]. One study, carried out by Minard et al. [64], demonstrated that in *Ae. albopictus*, the species *Acinetobacter baumannii* and *Acinetobacter johnsonii* may be involved in the blood digestion process. *Pantoea* was identified in a metagenomic analysis of the species *Ae. aegypti* [23] and *Cx. quinquefasciatus* [59]. The genus *Cellulosimicrobium* was isolated only in the BZIKV group. This genus was recently described by Schuman et al. [65], and was isolated from the intestine of the termite *Mastotermes darwiniensis* [66] and *Ae. albopictus* [67].

Part of the microbiota in adult mosquitoes acquired from the larval habitat and another fraction is dependent on food resources, whether they are fed sucrose or blood (in the case of females), and this significantly affects the diversity and abundance of the bacterial population [68]. The factors that may be related to bacterial diversity in the experimental groups in this study are the interactions between microbial communities and treatment with antibiotics. For example, *Serratia* and *Cedecea* spp. demonstrated co-exclusion relationships with dominant taxa, such as members of the genera *Asaia*, *Pseudomonas*, and *Enterobacter* in the microbiota of wild and colonized *Ae. aegypti*, *Ae. albopictus*, and *Cx. quinquefasciatus* [69]. Regarding antibiotic treatment, Gaio et al. [18] demonstrated that treatment of *Ae. aegypti* with antibiotics (carbenicillin, tetracycline, spectinomycin, gentamicin, and kanamycin) promoted a reduction in the abundance of cultivable bacteria; *Enterobacter* sp. and *Serratia* sp. were the predominant bacteria and were associated with hemolytic activity [18]. No reduction in abundance was observed; however, the antibiotic treatment did reveal the presence of *Enterobacter*, *Elizabethkingia*, *Pseudomonas*, and *Staphylococcus* [18].

To demonstrate the bacterial diversity described for *Aedes* spp. mosquitoes, we compared our results with the genera already identified in the current literature. We sought to demonstrate how the native microbiota could be modulated by the food source, and for this we compared the bacterial genera isolated in the unfed mosquitoes, mosquitoes fed with sucrose, blood, or blood infected with arbovirus (fed), and the isolated genera in this study (Additional file 1: Figure S1).

As shown in Additional file 1: Figure S1, the genera *Aeromonas*, *Elizabethkingia*, *Enterobacter*, *Pantoea*, *Pseudomonas*, and *Serratia* were identified in all established conditions. These findings suggest that, despite the modulation of the microbiota by the habitat and food source, there are bacteria that can adapt under the changing conditions of the holobiont [15, 23, 63].

Regarding the interaction between the mosquito, the virus, and the microbiota, we performed a reintroduction of bacteria in the *Ae. aegypti* treated with the pen/strep antibiotic in order to identify whether these bacteria could positively or negatively influence ZIKV infection. On the second day after feeding, a reduction in the intensity of infection was observed between the groups co-infected with ZIKV/*Aeromonas* and *Lysinibacillus*–*Lu. longipalpis* compared to the control groups (treated and untreated). On the seventh day, the reduction in viral load in the ZIKV/*Lysinibacillus*–*Ae. aegypti* co-infected group persisted, and we also observed a difference between the control group (treated) and the group co-infected with ZIKV/*Serratia*. In contrast, previous studies have revealed an association between *Serratia* sp. and increased infectivity and prevalence of DENV, ZIKV, and CHIKV in *Ae. aegypti* [26, 27, 46]. *Aeromonas* has also been associated with increased susceptibility to *Ae. aegypti* infection by DENV-2 [28]. Ramirez et al. [24] evaluated the influence of the reintroduction of *Proteus* and *Paenibacillus* on DENV infection in *Ae. aegypti*, and a decrease in the viral load of DENV was observed. At the taxonomic level, *Proteus* belongs to the family Enterobacteriaceae, and *Paenibacillus* belongs to the phylum Firmicutes, correlating with the genera *Serratia* (Enterobacteriaceae) and *Lysinibacillus* (phylum Firmicutes) evaluated in this study.

When comparing body and head-SG on the 14th day after feeding, we observed a significant reduction in viral load between the groups evaluated. It is known that the mosquito–virus interaction process can be influenced by anatomical barriers, digestive enzymes, the immune system, and the microbiota [70, 71]. The interaction between microbiota, immune system, and DENV in *Ae. aegypti* was demonstrated by Ramirez et al. [24]. It was observed that the reintroduction of isolated bacteria in the midgut of antibiotic-treated mosquitoes (pen/strep) caused changes in the abundance of antimicrobial peptide genes, including cecropins, gambicins, and attacins. The authors suggested that the modulation of the abundance of transcripts of the immunological gene by the reintroduced bacteria may have a nocuous effect on DENV infection [24].

Interestingly, the group with the highest number of viral copies in the head-SG was co-infected with the *Lysinibacillus*–*Lu. longipalpis* out-group, and the group with the lowest number of viral copies was the one co-infected with *Lysinibacillus*–*Ae. aegypti*. One possible explanation for this finding is the interaction between the insect, species vectors, and native microbiota. When *Lu. longipalpis* was co-infected with *Lysinibacillus* and *Leishmania* spp., a significant reduction in the parasitic load was observed [47]. By simultaneously analyzing viral abundance on the 14th dpi, we sought to assess the viability of ZIKV in co-infection via inoculation of infected SGs in the thorax of naïve mosquitoes. On the seventh dpi, it was possible to observe viable viruses in all the groups evaluated, with a median of around 10^9^ viral copies per mosquito. Secundino et al. [43] reported intrathoracic-inoculated *Ae. aegypti* (PP-Campos) with SGs infected with ZIKV viable viruses after 14 days, with the amount ranging from 2.7 × 10^6^ to 5.4 × 10^7^, and with a median of 1.49 × 10^7^ copies of ZIKV cDNA.

To demonstrate the impact of co-infection on transmission, the saliva of part of the infected mosquitoes (via the membrane) was analyzed. Salivation revealed a median of around 10^4^ copies in the control group pretreated with pen/strep, and around 10^3^ in the group co-infected with *Aeromonas*. Depending on the virus, mosquito species, and the quantification method employed, the estimated level of virus inoculated in mosquito saliva ranges from approximately 10^1^ to 10^7^ PFU [72, 73]. Consequently, mosquitoes co-infected with ZIKV/*Lysinibacillus*–*Ae. aegypti*, ZIKV/*Lu. longipalpis* did not present viable virus in their saliva, even though the virus was detected in the head-SG, as shown in Fig. 3. As demonstrated by other researchers, the detection of ZIKV in the saliva is a potential indicator of transmission to the host [74, 75].

## Conclusions

In summary, we demonstrate that the physiological conditions assessed herein influenced the composition of bacterial diversity. In the co-infection, among ZIKV, *Ae. aegypti* and the bacterial isolates, the ZIKV/*Lysinibacillus*–*Ae. aegypti* group had the lowest number of viral copies in the head-SG, which means that it negatively affected the vectorial competence. However, when the saliva was analyzed after forced feeding, no virus was detected in the mosquito groups ZIKV/*Lysinibacillus*–*Lu. longipalpis* or *Ae. aegypti*, and was partially detected in only one mosquito (ZIKV/*Serratia*). These results indicate that the combinations do not allow the development of viable viruses and thus show that they hold important potential as a biological control tool.

## Supplementary Information

**Additional file 1: Figure S1.** Bacterial diversity of *Aedes* spp. under different distinct feeding aspects [53, 56, 58, 59, 64, 68, 76–81]. Purple: unfed; green: fed (sucrose, blood, or blood infected with arbovirus); blue: isolated genera in this study. The Circos plot was generated using an online tool. http://circos.ca.

## Data Availability

The microbiota is a huge project still in analysis. Additional data is available from the corresponding author on reasonable request.

## Abbreviations

UF: Fed on sucrose, i.e., non-blood-fed
BF: Fed with non-infected blood
BZIKV: Fed with blood infected with ZIKV
TBF: Pretreated with pen/strep and fed with non-infected blood
TGZIKV: Pretreated with pen/strep and fed with blood infected with ZIKV, i.e., gravid with developed ovaries
dpi: Days post-infection
