# Supplementary figures and images for: The influence of culture-dependent native microbiota in Zika virus infection in Aedes aegypti
Source: Parasit Vectors. 2022 Feb 17;15:57. doi: 10.1186/s13071-022-05160-7 (PMC8851793; doi:10.1186/s13071-022-05160-7)

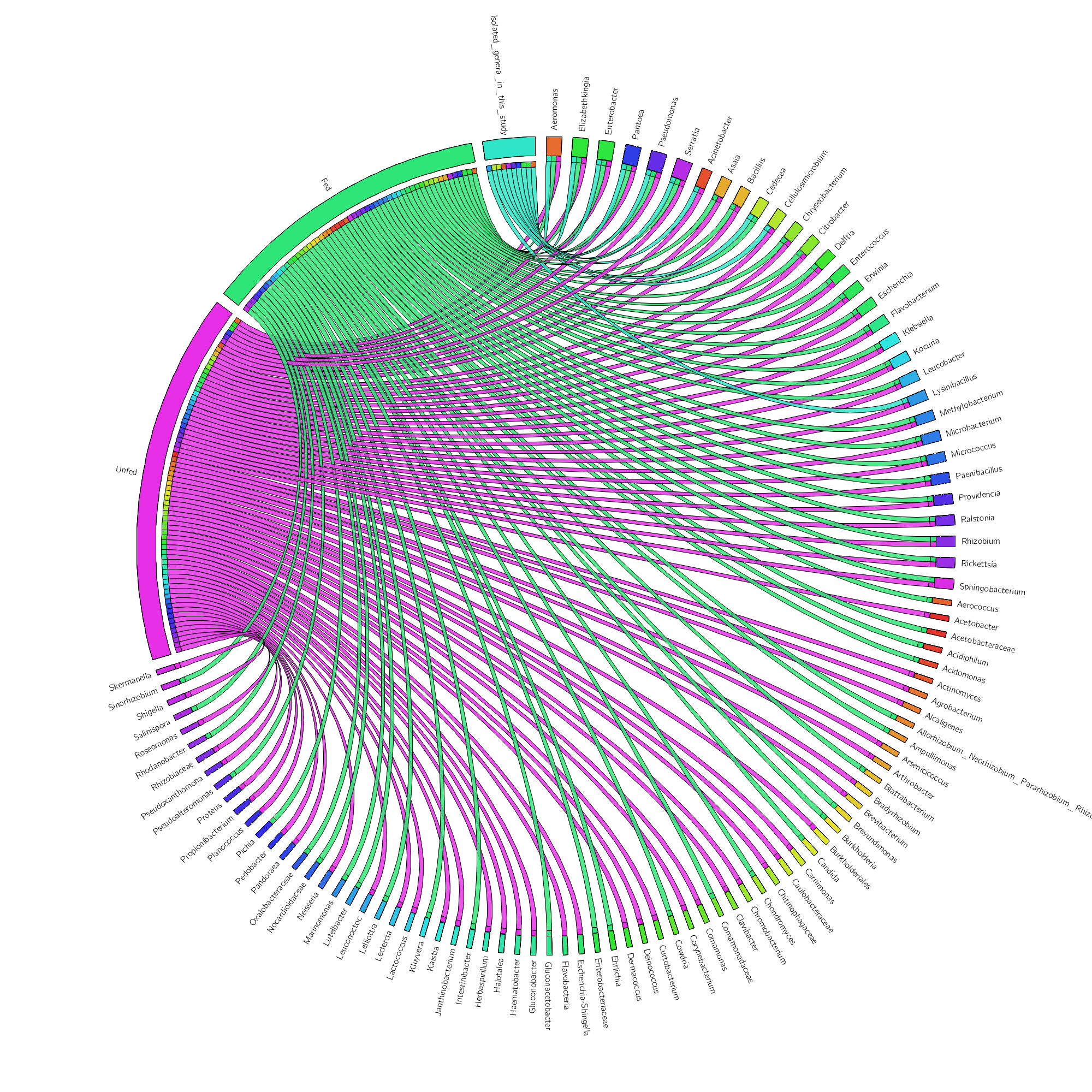

Supplement: Supplementary file 1 — Additional file 1: Figure S1. Bacterial diversity of Aedes spp. under different distinct feeding aspects [53, 56, 58, 59, 64, 68, 76–81]. Purple: unfed; green: fed (sucrose, blood, or blood infected with arbovirus); blue: isolated genera in this study. The Circos plot was generated using an online tool. http://circos.ca. [file 13071_2022_5160_MOESM1_ESM.png]
